# Outcomes of Total Hip Arthroplasty After Childhood Septic Hip Arthritis: A Systematic Review and Meta-Analysis of Infection Risk and Surgical Complications

**DOI:** 10.3390/children13040564

**Published:** 2026-04-18

**Authors:** Martina Ilardo, Marco Sapienza, Claudia de Cristo, Maria Agata Musumeci, Paola Torrisi, Noemi Di Paola, Alessia Caldaci, Andrea Vescio, Federico Canavese, Vito Pavone, Gianluca Testa

**Affiliations:** 1 Department of General Surgery and Medical Surgical Specialties, Section of Orthopedics, A.O.U. Policlinico “G. Rodolico-San Marco”, University of Catania, 95123 Catania, Italy; martinailardo52@gmail.com (M.I.); marcosapienza09@yahoo.it (M.S.); decristo.claudia@gmail.com (C.d.C.); musumeci.maria.mm@gmail.com (M.A.M.); paolatorrisi50@gmail.com (P.T.); dipaolanoemi@gmail.com (N.D.P.); alessia.c.92@hotmail.it (A.C.); andreavescio88@gmail.com (A.V.); vitopavone@hotmail.com (V.P.); 2 Orthopedic and Traumatology Department, IRCCS Istituto Giannina Gaslini, 16147 Genoa, Italy; canavese_federico@yahoo.fr

**Keywords:** septic arthritis, pediatric hip, total hip arthroplasty, periprosthetic joint infection, reinfection, revision, complications

## Abstract

**Background**: Total hip arthroplasty (THA) for the late sequelae of childhood septic hip arthritis is technically demanding, and infection-related risk remains incompletely defined. This systematic review and meta-analysis address the research question: “In adults undergoing THA after childhood septic arthritis of the hip, what is the incidence of post-THA infection, revision, and mechanical/neurologic complications?” We systematically reviewed and meta-analyzed outcomes after THA in patients with septic hip arthritis diagnosed at ≤18 years. **Methods**: PubMed, Web of Science, Scopus, and the Cochrane Library were searched from inception to 31 December 2025 (PRISMA). Eligible studies reported THA outcomes after childhood septic arthritis and met a Methodological Index for Non-Randomized Studies (MINORS) threshold (≥9). A random-effects meta-analysis of events per hip was performed. **Results**: Nine studies were included; eight contributed to the quantitative synthesis (343 hips). The pooled incidence of any post-THA infection was 1.55% (95% CI 0.38–3.48; *I*^2^ = 23.8%; 5/343); when microbiology was available, no relapse due to the index organism was reported and events were classified as new infections. The pooled incidence of revision for any cause was 4.99% (95% CI 2.27–8.70; *I*^2^ = 43.4%; 15/334). Non-infectious complications were clinically relevant, including intraoperative fracture (6.95%) and nerve palsy (4.84%). Evidence was limited by retrospective designs and heterogeneous reporting. **Conclusions**: THA after childhood septic hip arthritis demonstrates a low risk of postoperative infection, with relapse of the original pathogen appearing rare in carefully selected quiescent cases, but a clinically meaningful burden of mechanical and neurologic complications. These findings underscore the importance of careful preoperative assessment, meticulous surgical technique, and highlight the limitations of the current evidence. The protocol was registered in PROSPERO (ID: CRD420261298181). No external funding was received.

## 1. Introduction

Septic arthritis of the hip (SAH) in the pediatric population is an infrequent but highly aggressive condition that requires immediate orthopedic attention. Failure to promptly recognize and treat the infection may compromise joint integrity and lead to serious consequences, including irreversible femoral head damage, hip instability, growth disturbances, and premature degenerative joint disease [1,2]. Overall, pediatric septic arthritis (SA) is estimated to occur in approximately 1–10 per 100,000 children, and the hip joint appears to be the most commonly affected site [1,3,4]. Despite this, the true incidence of SAH is poorly defined, largely due to diagnostic uncertainty and overlap with other pediatric hip disorders [5].

Diagnosis relies on a combination of clinical assessment, laboratory evaluation, predictive scoring systems, and imaging studies [5,6,7]. Several clinical prediction models have been developed to support diagnostic decision-making, among which Kocher’s criteria are the most widely applied [6]. Nevertheless, none of these tools alone can definitively establish a diagnosis. Joint aspiration with synovial fluid analysis and microbiological culture remains the gold standard to confirm infection and guide antimicrobial therapy [1,6]. Early intervention is critical to preserve joint structure and function. Management of SAH typically involves a combination of surgical drainage and antibiotic therapy. Previous studies emphasize that prompt joint decompression combined with targeted antibiotic treatment is essential to eradicate infection, prevent cartilage destruction, and reduce the risk of long-term sequelae such as growth disturbance and joint deformity [1,5,6,8,9]. When diagnosis or treatment is delayed, SAH may result in long-lasting morbidity, including impaired hip development, functional limitations, and increased healthcare burden [10,11,12]. Residual deformities may involve both the femoral head and acetabulum, leading to instability, subluxation, or dislocation and, ultimately, secondary degenerative joint disease. Several classification systems have been introduced to describe post-septic sequelae and guide treatment strategies. Hunka et al. developed a system based on residual deformity and functional impairment [13], while Forlin and Milani proposed a classification emphasizing anatomical alterations and treatment options [14]. Johari et al. described a system based on the presence of the capital femoral epiphysis and hip stability [15]. The classification proposed by Choi et al. focuses on radiographic patterns of deformity and their surgical implications [16]. More recently, the HHPO classification has been introduced to further refine the assessment of post-septic hip sequelae [10]. Age at infection is a major determinant of prognosis. Infection during infancy may disrupt epiphyseal development and growth, resulting in deformity, whereas timely treatment after epiphyseal maturation may allow near-normal development. Nonetheless, growth injury can result in coxa breva, coxa vara, coxa magna, proximal femoral deformity, angular deformities, limb-length discrepancy, dislocation, pseudarthrosis, or ankylosis [16,17,18,19,20,21].

When end-stage degeneration develops and conservative measures fail, total hip arthroplasty (THA) is often considered the definitive procedure to relieve pain and restore function [22]. However, THA after post-septic sequelae is frequently considered to carry higher risk than routine primary arthroplasty [23]. Patients are often young and present with dysplastic acetabula, proximal femoral deformity, compromised bone stock, and severe soft-tissue contractures, all of which increase technical difficulty and may affect implant survival and revision risk. In addition, scarring and contractures can hinder exposure, alter soft-tissue tension, and complicate restoration of hip biomechanics [23].

Prior reconstructive surgery and retained hardware may further increase procedural complexity and contribute to incomplete functional recovery [23,24]. Reported complications in this subset include traction-related nerve injury (e.g., sciatic palsy), intraoperative or periprosthetic fractures, limb-length discrepancy, instability, and mechanical loosening [22,23,25]. Periprosthetic joint infection (PJI) is uncommon even after standard primary THA (estimated 0.4–2.2%) [26,27], but there is persistent concern that arthroplasty after childhood SA may carry a higher risk of infection-related failure, even when performed after apparent infection control [27].

A prolonged symptom-free quiescent interval of more than 10 years has been advocated by some authors to reduce the likelihood of persistent or occult infection and optimize outcomes [23,27]. Moreover, achieving stable reconstruction and minimizing neurovascular risk may require additional reconstructive procedures such as bone grafting, use of small acetabular components, complex femoral preparation for hypoplasia or femoral bowing, and, in selected cases, subtrochanteric shortening osteotomy with extensive soft-tissue release [25,27].

Given the limited and heterogeneous evidence available for this specific population and the variability in reported outcomes, the aim of this study was to systematically review the literature and quantitatively synthesize outcomes after total hip arthroplasty (THA) in patients with a pediatric history of septic arthritis of the hip.

## 2. Materials and Methods

### 2.1. Search Strategy

This systematic review and meta-analysis was conducted in accordance with the PRISMA 2020 statement. The review protocol was registered in PROSPERO (ID: CRD420261298181). No protocol amendments were made. The full protocol is available in the PROSPERO record.

A literature search was performed in four databases (PubMed, Web of Science, Scopus, and the Cochrane Library) from inception to 31 December 2025. These databases were selected to ensure comprehensive coverage of the literature: PubMed as the primary biomedical database, Web of Science and Scopus to broaden retrieval and capture additional peer-reviewed studies and citation data, and the Cochrane Library to identify relevant systematic reviews and controlled studies. The search strategy combined keywords and Boolean operators as follows: (“septic arthritis hip” OR “septic arthritis of the hip” OR “SAH”) AND (“total hip arthroplasty” OR “total hip replacement” OR “THA” OR “hip prosthesis”) AND (“outcomes” OR “complications” OR “infection” OR “revision”). Reference lists of eligible articles were screened to identify additional studies. The full electronic search strategies for all sources are provided in the Supplementary Materials. We did not perform a formal search of grey literature sources (e.g., conference proceedings, theses, or trial registries), and only peer-reviewed articles indexed in the selected databases were considered. Two reviewers (M.I. and M.M.) independently screened titles/abstracts and then full-text articles for eligibility; disagreements were resolved by consensus.

### 2.2. Inclusion and Exclusion Criteria

The inclusion criteria were as follows: (1) retrospective and prospective studies on conservative or surgical treatments of SAH; although the initial search captured both conservative and surgical management, only studies reporting THA outcomes were included in the quantitative synthesis and meta-analysis (2) patients aged ≤18 years at the time of infection; (3) incidence of THA in patients treated for SAH; (4) articles published in English; (5) full article text available; and (6) studies with a Methodological Index for Non-Randomized Studies (MINORS) quality evaluation score ≥9 points. The exclusion criteria were as follows: (1) case reports and reviews; (2) studies from which the incidence of THA could not be extracted; (3) other hip disorders, such as slipped capital femoral epiphysis, femoral neck fracture, and Legg–Calvé–Perthes disease; and (4) studies with MINORS quality evaluation score <9 points.

### 2.3. Quality Evaluation

The quality of the included studies was assessed using the MINORS quality evaluation criteria: clearly stated aim, inclusion of consecutive patients, prospective data collection, endpoints appropriate to the aim of the study, unbiased assessment of the study endpoint, follow-up period appropriate to the aim of the study, loss to follow-up <5%, prospective calculation of the study size, adequate control group, temporary groups, baseline equivalence of groups, and adequate statistical analysis. The latter four items apply exclusively to comparative studies, and the first eight apply to both comparative and non-comparative studies. The items were scored as 0 (not reported), 1 (reported but inadequate), or 2 (reported and adequate). The MINORS tool was selected as it is specifically designed and validated for the assessment of non-randomized studies, including retrospective case series, which constituted the entirety of the included evidence. A minimum threshold of ≥9 was applied to exclude studies with very low methodological quality while retaining a sufficient number of studies for analysis, representing a balance between methodological rigor and feasibility given the limited available data. Two researchers independently scored the studies according to the MINORS criteria. In the event of a conflicting evaluation of an article, the two evaluators proposed a common score after discussion.

### 2.4. Outcomes

The primary quantitative outcome was any post-THA infection, defined as the occurrence of infection after THA in patients with a history of septic arthritis. Three distinct infection outcomes were defined for this review:

(1) Any post-THA infection—all infections occurring after THA, regardless of microbiology;
(2) Relapse/recurrence—infections caused by the same microorganism as the index septic arthritis episode;
(3) New infection—infections caused by a different microorganism.

For the purpose of this review, a “quiescent infection” was defined as the absence of active infection at the time of THA, based on a combination of the following criteria when reported: (1) a prolonged infection-free interval from the index septic arthritis episode; (2) absence of clinical signs of infection (e.g., pain, fever, sinus tract); (3) normal inflammatory markers (e.g., ESR, CRP, white blood cell count); and/or (4) negative microbiological investigations, including joint aspiration or intraoperative cultures. Given the heterogeneity across studies, no single criterion was required for inclusion, and the definition was based on the criteria reported by each individual study.

Secondary outcomes included revision for any cause and specific complications after THA (dislocation, periprosthetic fracture, intraoperative fracture, nerve palsy, and venous thromboembolism [VTE]). In addition, we extracted study-level characteristics (e.g., country, study design, sample size, age at THA, quiescent interval, fixation strategy and adjunctive procedures, and follow-up duration). Outcome event counts were extracted at the latest reported follow-up. Data extraction was performed by one reviewer and checked by a second reviewer; disagreements were resolved by consensus. Each hip was considered as an independent observation. Some patients contributed two hips. Denominators differ between outcomes because only studies reporting a given complication were included. Non-reporting was not assumed to indicate zero events. Overall certainty of evidence for each outcome (any infection, reinfection/relapse, revision, and major complications) was rated as low or very low due to retrospective design, small sample sizes, and rarity of events.

### 2.5. Statistical Analysis

A meta-analysis of the rate of events per hip was performed for each endpoint when at least two studies reported the outcome and provided extractable event counts. Studies that did not report a given endpoint were excluded from that synthesis and were not assumed to have zero events. Given the expected clinical heterogeneity (differences in patient characteristics, surgical technique, and follow-up), pooled estimates were calculated using a random-effects model and displayed using forest plots. Events per hip were pooled using the Freeman–Tukey double arcsine transformation, which is robust for rare events and accommodates studies with zero events.

Between-study variance was estimated with the DerSimonian–Laird method. Study-level 95% confidence intervals (CIs) were computed using the Clopper–Pearson exact method. Heterogeneity was assessed with Cochran’s Q and quantified using *I*^2^. A weighted mean follow-up (weighted by the number of hips) was calculated for the main endpoints. All analyses were conducted using a dedicated script in Python (version 3.x, Python Software Foundation, Wilmington, DE, USA). Due to the limited number of studies contributing to each outcome, formal assessment of small-study effects/publication bias (e.g., funnel plots or Egger’s test) was not performed. The certainty of evidence was assessed following the GRADE approach guidelines to provide a transparent summary of the quality of findings. Sensitivity and subgroup analyses were not performed due to the limited number of studies per outcome.

## 3. Results

### 3.1. Search Results

Our search strategy identified a total of 5813 records. After removal of, 2388 duplicates, 3425 articles were screened. Of these, 2103 studies were excluded based on title screening, and another 1249 were excluded after abstract review. Nine articles were subsequently excluded because the full text was not available.

**Figure 1:**
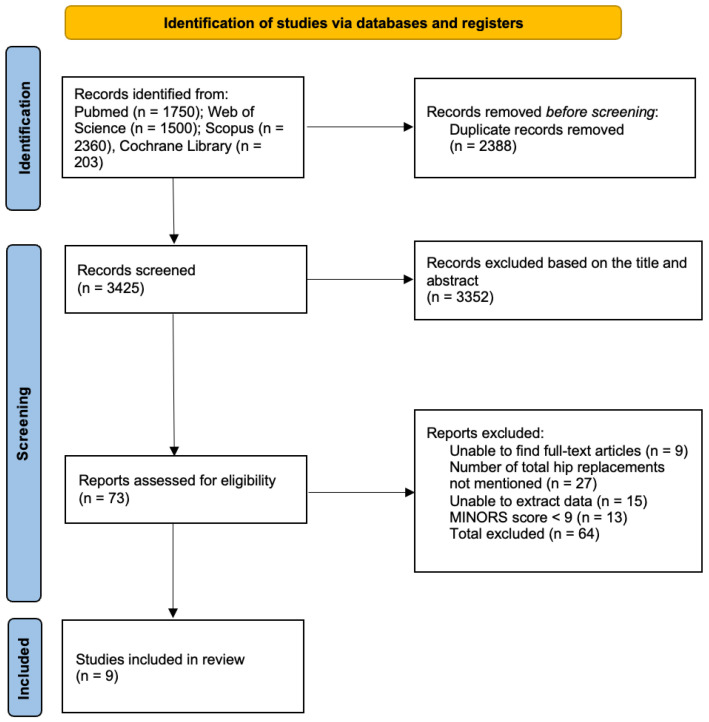
PRISMA flow diagram of study selection. The flowchart illustrates the stages of identification, screening, and eligibility assessment. From the initial 5813 records identified through PubMed, Web of Science, Scopus, and the Cochrane Library, 9 studies met the final inclusion criteria for the systematic review. Specific reasons for exclusion at the full-text level (e.g., MINORS score < 9, insufficient data) are detailed in the corresponding boxes.

Of the remaining studies assessed for eligibility, 27 were excluded because the incidence of THA was not reported or could not be extracted, 15 could not be assigned to conservative or surgical treatment groups, and 13 failed to meet the predefined quality scoring criteria. Nine studies were included in the systematic review; eight contributed data to the quantitative synthesis, whereas one study was included in the qualitative synthesis only. The characteristics of the studies in the meta-analysis are summarized in Table 1, and the PRISMA flow diagram is shown in Figure 1.

### 3.2. Post-THA Infection (Primary Outcome)

**Figure 2:**
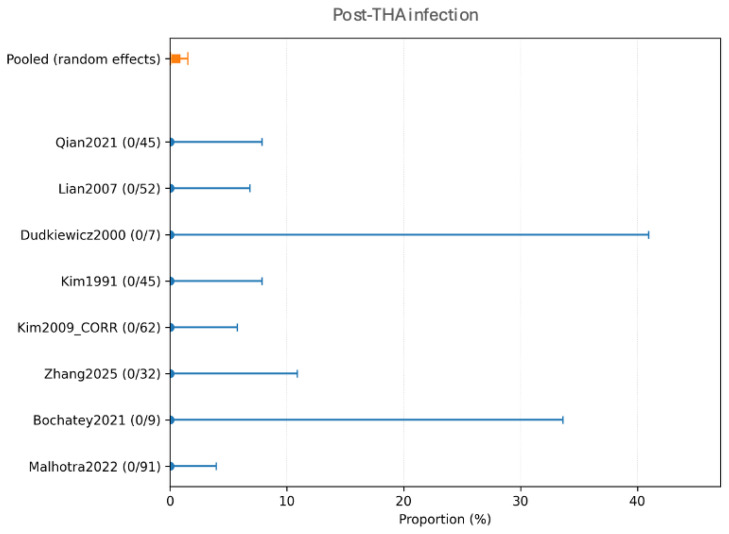
Forest plot of any post-THA infection (including both new infections and relapse/recurrence). Random-effects meta-analysis of events per hip. A total of k = 8 studies and N = 343 hips were included. The pooled estimate was 1.55% (95% CI 0.38–3.48), with heterogeneity *I*^2^ = 23.8% [23,25,28,29,30,31,32,33]. Regarding the graphical representation, blue circles and horizontal lines indicate point estimates and 95% confidence intervals for individual studies, while the orange square represents the pooled proportion.

Eight studies (343 hips in total) contributed to the quantitative synthesis of post-THA infection. The pooled incidence of any post-THA infection was 1.55% (95% CI 0.38–3.48%) with low-to-moderate heterogeneity (*I*^2^ = 23.8%), which corresponds to 5 events of infection with the same organism as the index episode of SA. It should be noted that these events were not relapses of the original septic arthritis pathogen; when microbiological information was available, all infections were classified as new infections caused by different organisms, distinct from true reinfection/relapse. The weighted mean follow-up for this endpoint was 7.84 years (Figure 2).

### 3.3. Revision for Any Cause

**Figure 3:**
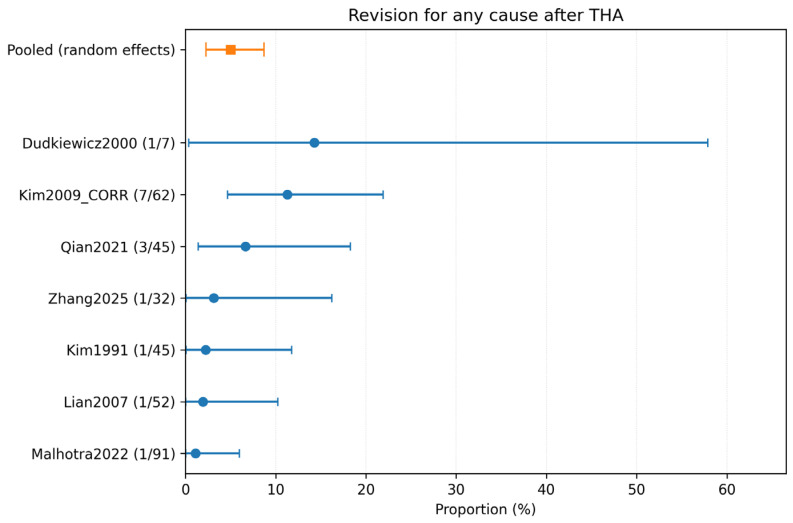
Forest plot of revision for any cause after THA. Random-effects meta-analysis of events per hip. A total of k = 7 studies and N = 334 hips were included. The pooled estimate was 4.99% (95% CI 2.27–8.70), with heterogeneity *I*^2^ = 43.4% [23,25,29,30,31,32,33]. Regarding the graphical representation, blue circles and horizontal lines indicate point estimates and 95% confidence intervals for individual studies, while the orange square represents the pooled proportion.

Seven studies (334 hips) reported revision outcomes. The pooled incidence of revision for any cause was 4.99% (95% CI 2.27–8.70%) with moderate heterogeneity (*I*^2^ = 43.4%), which corresponded to 15 revisions across 334 hips. The weighted mean follow-up time for revision analysis was 7.97 years (Figure 3).

### 3.4. Complication-Specific Meta-Analyses

**Figure 4:**
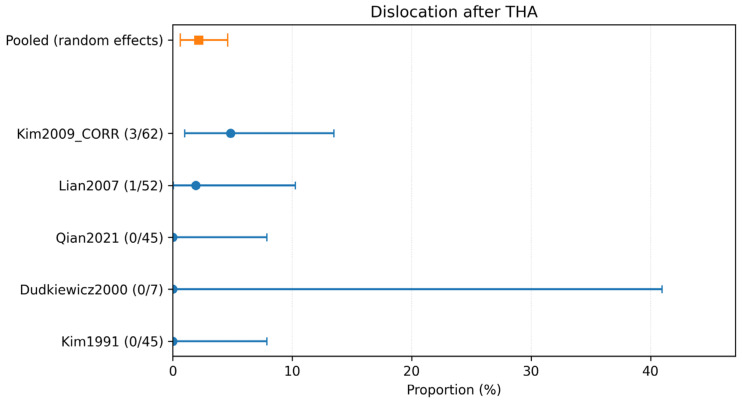
Forest plot of dislocation after THA. Random-effects meta-analysis of events per hip. A total of k = 5 studies and N = 211 hips were included. The pooled estimate was 2.18% (95% CI 0.64–4.60), with heterogeneity *I*^2^ = 2.3% [25,30,31,32,33]. Regarding the graphical representation, blue circles and horizontal lines indicate point estimates and 95% confidence intervals for individual studies, while the orange square represents the pooled proportion.

**Figure 5:**
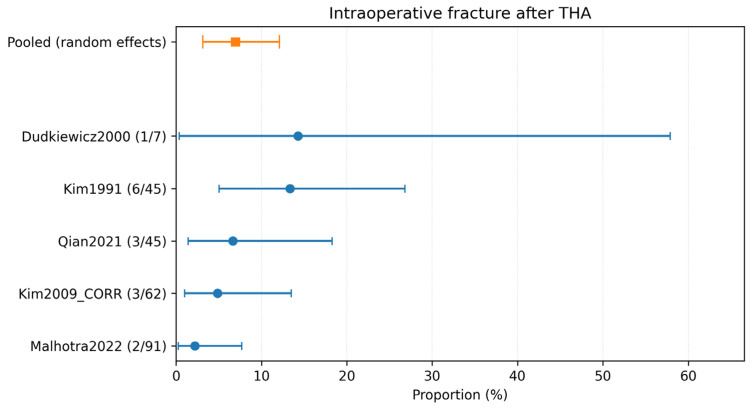
Forest plot of intraoperative fracture after THA. Random-effects meta-analysis of events per hip. A total of k = 5 studies and N = 250 hips were included. The pooled estimate was 6.95% (95% CI 3.14–12.11), with heterogeneity *I*^2^ = 45.1% [23,25,30,31,32]. Regarding the graphical representation, blue circles and horizontal lines indicate point estimates and 95% confidence intervals for individual studies, while the orange square represents the pooled proportion.

**Figure 6:**
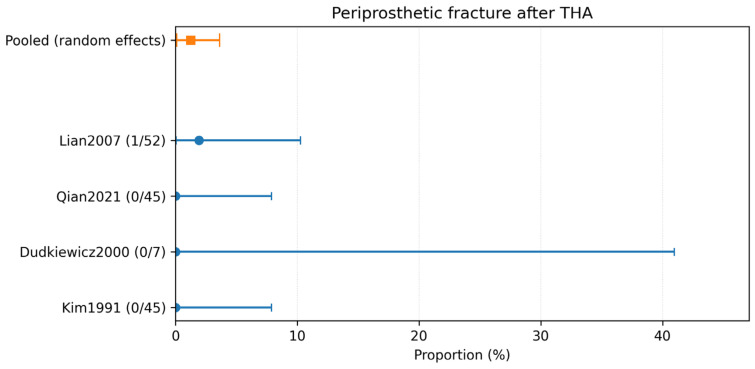
Forest plot of periprosthetic fracture after THA. Random-effects meta-analysis of events per hip. A total of k = 4 studies and N = 149 hips were included. The pooled estimate was 1.24% (95% CI 0.10–3.61), with heterogeneity *I*^2^ = 0% [25,31,32,33]. Regarding the graphical representation, blue circles and horizontal lines indicate point estimates and 95% confidence intervals for individual studies, while the orange square represents the pooled proportion.

**Figure 7:**
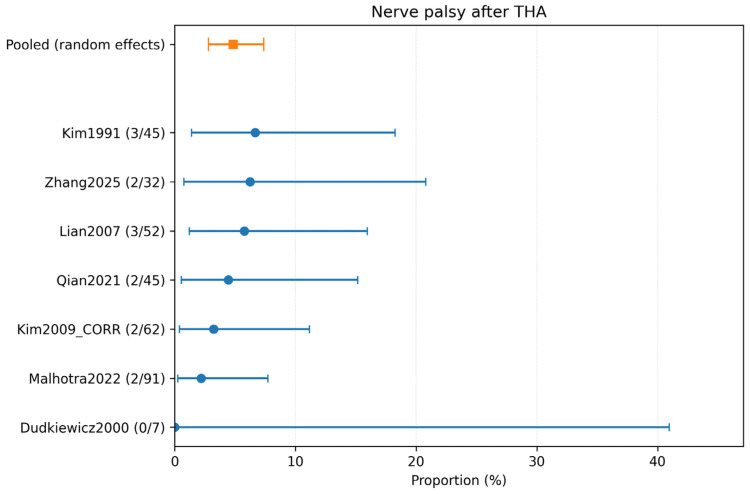
Forest plot of nerve palsy after THA. Random-effects meta-analysis of events per hip. A total of k = 7 studies and N = 334 hips were included. The pooled estimate was 4.84% (95% CI 2.81–7.38), with heterogeneity *I*^2^ = 0% [23,25,29,30,31,32,33]. Regarding the graphical representation, blue circles and horizontal lines indicate point estimates and 95% confidence intervals for individual studies, while the orange square represents the pooled proportion.

Complication-specific pooled estimates were calculated using only studies explicitly reporting each outcome. The pooled incidence of dislocation was 2.18% (95% CI 0.64–4.60%; 5 studies, 211 hips; *I*^2^ = 2.3%) (Table 2; Figure 4). The pooled incidence of intraoperative fracture was 6.95% (95% CI 3.14–12.11%; 5 studies, 250 hips; *I*^2^ = 45.1%) (Table 2; Figure 5). The pooled incidence of periprosthetic fracture was 1.24% (95% CI 0.10–3.61%; 4 studies, 149 hips; *I*^2^ = 0%) (Table 2; Figure 6). The pooled incidence of nerve palsy was 4.84% (95% CI 2.81–7.38%; 7 studies, 334 hips; *I*^2^ = 0%) (Table 2; Figure 7). Venous thromboembolism (VTE) was reported in three studies, comprising fewer than 100 hips, and therefore was not included in the pooled complication analysis (Table 2). A summary of the pooled absolute risks and the certainty of the evidence (GRADE) for each outcome is reported in Table 3.

## 4. Discussion

Childhood SAH can result in substantial long-term sequelae, including residual deformity, acetabular dysplasia, soft-tissue contractures, limb-length discrepancy, and premature osteoarthritis, eventually requiring THA in young adulthood [33,34]. Our quantitative analysis suggests “dual profiles” of outcomes: low risk of infection-related failure and a clinically meaningful burden of technical non-infectious complications. These profiles are consistent with prior syntheses on THA for post-infectious childhood hip sequelae [27,34].

In the quantitative synthesis, eight studies (343 hips) contributed to the primary endpoint. The pooled incidence of any post-THA infection, including new infections, was 1.55% (95% CI 0.38–3.48%), with low-to-moderate heterogeneity (*I*^2^ = 23.8%). Moreover, the pooled estimate for relapse/recurrence of the index infection (“reinfection”) was very low (0.51%; 95% CI 0.04–1.53; *I*^2^ = 0%), which is consistent with the absence of observed relapse events in the included cohorts. Importantly, when microbiological information was available, no event consistent with relapse/recurrence due to the same organism as the index episode was reported, and recorded events were classified as new infections (different organism).

A central interpretative issue in arthroplasty after SA is whether postoperative infections represent relapse of the index episode or a new infection occurring after implantation. In the included cohorts, cases were predominantly managed in a quiescent setting, often after prolonged symptom-free intervals, and relapse of the index infection was not observed [23,26,28]. However, the definition of “quiescent” was not standardized across studies and was based on variable combinations of clinical, laboratory, and microbiological criteria, which may limit comparability across cohorts. In contrast, a small number of postoperative infections were recorded as new infections when organism information was available, supporting the concept that late infections after THA may not necessarily reflect reactivation of the original SA, but rather new infectious episodes occurring after implantation. The infection risk is not completely abolished even after successful eradication of the initial SA [23,28].

This concept is consistent with contemporary frameworks for PJI, where standardized diagnostic criteria (e.g., MSIS/ICM-derived definitions) are recommended to improve comparability and reduce misclassification across studies. In addition, infectious disease guidelines emphasize the need for a structured diagnostic approach integrating clinical features, laboratory markers, and microbiology in suspected PJI [35,36]. In line with these principles, several included series were performed after long quiescent intervals prior to THA. This strategy is commonly advocated to reduce the risk of persistent or occult infection and to optimize candidate selection [23,27,28].

Nevertheless, a low relapse rate should not be interpreted as “zero infection risk,” particularly because microbiological reporting and diagnostic work-up are inconsistent across cohorts. Even in conventional primary THA, PJI is an ongoing burden and may be underestimated if registry data are used in isolation [37]. In addition, comparative evidence suggests that patients undergoing THA after prior to SA may have higher risks of infection-related outcomes than patients undergoing THA for primary osteoarthritis. Case–control studies have reported increased risks of PJI or any infection in a post-septic setting compared with matched osteoarthritis controls, and some data suggest that infection risk decreases as the interval between SA and THA increases [38,39]. Although these comparative cohorts are not directly inter-changeable with the predominantly childhood-sequelae populations in our meta-analysis, they reinforce the need for careful preoperative counseling and standardized assessment of infection risk.

Despite the reassuring infection signal, revision and complication rates highlight the technical challenge of THA in anatomically distorted post-infectious hips. The pooled revision rate for any cause was 4.99% (95% CI 2.27–8.70; *I*^2^ = 43.4%; 15/334 hips; 7 studies). Complication-specific pooled estimates highlighted intraoperative fracture (6.95% (95% CI 3.14–12.11; *I*^2^ = 45.1%; 15/250 hips; 5 studies)) and nerve palsy (4.84% (95% CI 2.81–7.38; *I*^2^ = 0%; 14/334 hips; 7 studies)) as the most frequent events, followed by dislocation (2.18% (95% CI 0.64–4.60; *I*^2^ = 2.3%; 4/211 hips; 5 studies)), periprosthetic fracture (1.24% (95% CI 0.10–3.61; *I*^2^ = 0%; 1/149 hips; 4 studies)), and VTE (1.70% (95% CI 0.10–5.17; *I*^2^ = 0%; 1/97 hips; 3 studies)). These findings are clinically plausible because THA after childhood infection often resembles a complex reconstructive procedure rather than a straightforward primary arthroplasty.

Residual acetabular deficiency, narrow femoral canals, altered femoral version, and dense scar tissue can increase the risk of iatrogenic fracture, instability, and traction-related nerve injury, as well as the frequent need for extensive releases and adjunctive procedures [23,25,27,29,30,31,32,33]. Malhotra et al. emphasize that operating on hips with septic sequelae is technically demanding and has been associated with complications such as intraoperative femoral fractures and nerve injury, with subtrochanteric osteotomy and extensive soft-tissue releases that are often required to facilitate reduction and mitigate neurovascular risk [23,28]. Similarly, in a mid-term series using circumferential medial acetabular wall osteotomy, Lian et al. reported traction-related femoral and sciatic nerve injuries, as well as instability and fracture-related events, which underscore the vulnerability of bone stock and soft tissues in this population [33,34]. Other series describing high dislocation and severe deformity after childhood infection further support the concept that these procedures often behave more like reconstructive surgery than straightforward primary arthroplasty [25,28,29,30,31,32].

From a clinical perspective, our results support a balance between infection prevention strategies and mitigation of technical complications. Preoperative assessment aimed at excluding persistent or occult infection has been advocated, including synovial fluid cultures when indicated, use of intraoperative diagnostic adjuncts (e.g., frozen sections) to increase the likelihood of detecting silent infection, and tailoring postoperative antibiotic therapy [35]. At the same time, prevention of mechanical and neurologic complications should be prioritized through meticulous templating, judicious soft-tissue release, and consideration of strategies to reduce fracture and traction-related nerve injury (e.g., preventive cerclage cabling, a lower threshold for shortening osteotomy in selected cases, appropriate implant options for narrow canals). Finally, although not included in the quantitative synthesis, Benum’s long-term experience with trochanteric apophyseal transposition illustrates that joint-preserving or reconstructive procedures in childhood can delay end-stage degeneration and may create more favorable conditions for later hip replacement. This reinforces the life-course trajectory from infection to reconstruction to arthroplasty [40].

This study has limitations that should be considered when interpreting the pooled estimates. First, the evidence base consisted almost exclusively of retrospective observational studies and case series, which are susceptible to selection bias (including preferential inclusion of quiescent cases), incomplete outcome capture, and variable follow-up. Second, clinical and methodological heterogeneity was substantial and reflect differences in baseline deformity severity, surgical techniques (including cemented vs. cementless fixation and use of osteotomy), age at THA, which may influence postoperative outcomes and complication rates, quiescent interval, and follow-up duration. This heterogeneity likely contributed to between-study variability for revision, intraoperative fracture, and nerve palsy and precluded formal subgroup or sensitivity analyses. The use of a MINORS ≥9 threshold ensured inclusion of methodologically robust studies; however, it may have excluded smaller cohorts that could contribute valuable descriptive data, a limitation inherent to systematic reviews in rare conditions.

Infection-related definitions and diagnostic work-up were inconsistently reported, and microbiological data regarding the index infection and postoperative infections were not uniformly available, which limited pathogen-informed interpretation and the ability to robustly classify relapse versus new infection across all cohorts. Several complication endpoints were reported by only a subset of studies, and non-reported outcomes could not be assumed to be true zero-event data. The pooled incidence of venous thromboembolism (VTE) was reported in only three studies including fewer than 100 hips and was therefore not included in the pooled complication analysis, limiting conclusions on thromboembolic risk. In addition, heterogeneity across studies regarding calendar period, age at THA, interval between SAH and arthroplasty, surgical technique, fixation method, and reconstructive strategy may influence both infection risk and the incidence of mechanical and neurological complications. Due to the small number of studies, sensitivity analyses excluding early series or those with very short follow-up could not be performed, which should be considered a limitation. Although the pooled incidence of postoperative infection was low, the small number of events and wide confidence intervals highlight the need for caution when interpreting these results, particularly for patients with shorter quiescent intervals or less selected cohorts. Importantly, registry-based and large cohort data specifically addressing THA after childhood septic arthritis of the hip are currently lacking. The search strategy did not include grey literature sources, which may have led to the omission of relevant unpublished or non-indexed studies. Given the rarity of this condition and the low incidence of infection-related events, future research would benefit from multicentre collaboration and, where feasible, linkage with national arthroplasty registries. Such approaches may improve statistical power, enable more accurate estimation of rare complications, and provide insights into long-term outcomes in this clinically challenging population.

## 5. Conclusions

Total hip arthroplasty (THA) performed for the late sequelae of childhood septic arthritis of the hip showed a postoperative infection rate of 1.55%, with no documented relapse of the index infection when microbiological data were available. The overall revision rate was 4.99% at a mean follow-up of approximately 8 years.

The most frequent complications were intraoperative fracture (6.95%) and nerve palsy (4.84%), followed by dislocation (2.18%), periprosthetic fracture (1.24%), and venous thromboembolism (1.70%), indicating that non-infectious complications represent the main source of morbidity in this population. THA in this setting is technically demanding due to acetabular dysplasia, proximal femoral deformity, narrow femoral canals, altered femoral version, and soft-tissue contractures, which increase the risk of intraoperative fracture and traction-related nerve injury. Precautions to reduce complication risk include thorough preoperative assessment to exclude persistent or occult infection, detailed surgical planning with appropriate implant selection, careful soft-tissue balancing, and consideration of adjunctive procedures such as femoral shortening osteotomy or prophylactic cerclage in selected cases. At mid-term follow-up, THA provides infection control with a low rate of infection-related failure, but mechanical and neurologic complications remain frequent. The available evidence is limited to retrospective studies, with no prospective studies identified, and is characterized by heterogeneous reporting.

Further multicenter studies with standardized definitions of infection and complications are required to better define long-term outcomes and optimize surgical strategies.

## Figures and Tables

**Table 1:** Characteristics of studies included in meta-analysis. NR = not reported. Most studies described septic arthritis as occurring in childhood without providing specific age data.

Study	Design	Country/ Center	Hips (*n*)	Mean Age at THA (y)	Mean Follow-Up (y)	Age at SAH (y)	Interval SAH–THA (y)	Tsukuyama/ Zimmerli Classification	Pediatric Sequela	Minors
Malhotra (2022) [23]	Retrospective case series	AIIMS, New Delhi, India	91	29.1	6.50	5.2	23.9	Type II (healed)/ Sequelae	Yes	10
Bochatey (2021) [28]	Retrospective observational (quiescent)	British Hospital of Buenos Aires, Argentina	9	37.2	3.02	NR	18.8	Type II (healed)/ Sequelae	Yes	10
Zhang (2025) [29]	Retrospective observational	China (single center)	32	55.6	4.60	9.8	35.2	Type II (healed)/ Sequelae	Yes	9
Kim (2009) [30]	Retrospective case series	Korea (single center)	62	47.5	15.20	NR	37.8	Type II (healed)/ Sequelae	Yes	10
Kim (1991) [31]	Retrospective case series	Texas Tech University Health Sciences Center, USA	45	36.4	5.45	NR	19.9	Type II (healed)/ Sequelae	Yes	9
Dudkiewicz (2000) [32]	Case series	Chaim Sheba Medical Center, Tel Aviv, Israel	7	19.1	8.14	NR	NR	Type II (healed)/ Sequelae	Yes	10
Lian (2007) [33]	Retrospective case series	West China Hospital, Chengdu, China	52	44.5	7.80	NR	36.8	Type II (healed)/ Sequelae	Yes	11
Qian (2021) [25]	Retrospective case series	First Affiliated Hospital of Xinjiang Medical University, China	45	46.0	6.10	NR	38.2	Type II (healed)/ Sequelae	Yes	10

**Table 2:** Random-effects meta-analysis results (events per hip).

Outcome	*k*	Hips (*N*)	Events	Pooled Proportion (%)	95% CI (%)	*I*^2^ (%)
Any post-THA infection	8	343	5	1.55	0.38–3.48	23.8
Reinfection/relapse	8	343	0	0.51	0.04–1.53	0.0
Revision for any cause	7	334	15	4.99	2.27–8.70	43.4
Dislocation	5	211	4	2.18	0.64–4.60	2.3
Intraoperative fracture	5	250	15	6.95	3.14–12.11	45.1
Periprosthetic fracture	4	149	1	1.24	0.10–3.61	0.0
Nerve palsy	7	334	14	4.84	2.81–7.38	0.0

*k*, number of studies contributing to each meta-analysis. Reinfection after THA refers specifically to recurrence of the original pathogen responsible for the index septic arthritis. All other postoperative infections reported in the studies are considered new infections caused by different organisms. VTE reported descriptively only due to limited number of events and studies.

**Table 3:** Summary of findings and certainty of evidence (GRADE) for outcomes after total hip arthroplasty (THA) performed for late sequelae of childhood septic arthritis of the hip. GRADE (Grading of Recommendations, Assessment, Development and Evaluations) was used to assess the certainty of evidence. **a.** Downgraded one level for very serious risk of bias (primarily due to the retrospective nature of included studies and lack of control groups). **b.** Downgraded one level for serious imprecision (small cumulative sample size and wide confidence intervals). **c.** Downgraded one level for serious inconsistency (high heterogeneity, *I*^2^ > 40%). **d.** Downgraded one level for potential publication bias.

Outcome	Studies (Hips)	Follow-Up (Years)	Pooled Incidence % (95% CI)	*I*^2^ (%)	Certainty (GRADE)
Any post-THA infection (new or relapse)	8 (343)	7.84	1.55 (0.38–3.48)	23.8	Very low ^a,b,d^
Relapse/recurrence (same organism) after THA	8 (343)	7.84	0.51 (0.04–1.53)	0.0	Very low ^a,b,d^
Revision for any cause	7 (334)	7.97	4.99 (2.27–8.70)	43.4	Very low ^a,b,c^
Dislocation	5 (211)	Not consistently reported	2.18 (0.64–4.60)	2.3	Very low ^a,b^
Intraoperative fracture	5 (250)	Perioperative	6.95 (3.14–12.11)	45.1	Very low ^a,b,c^
Periprosthetic fracture	4 (149)	Not consistently reported	1.24 (0.10–3.61)	0.0	Very low ^a,b^
Nerve palsy	7 (334)	Not consistently reported	4.84 (2.81–7.38)	0.0	Low ^a^

## Data Availability

Not Applicable.

## Supplementary Materials

The following supporting information can be downloaded at: https://www.mdpi.com/article/10.3390/children13040564/s1, Detailed Search Strategy (as registered in PROSPERO).

## Abbreviations

The following abbreviations are used in this manuscript:

THA	Total Hip Arthroplasty
SAH	Septic Arthritis of the Hip
VTE	Venous Thromboembolism
PJI	Periprosthetic Joint Infection
